# *Dendrocalamus menghanensis* (Poaceae, Bambusoideae), a new woody bamboo from Yunnan, China

**DOI:** 10.3897/phytokeys.130.33948

**Published:** 2019-08-29

**Authors:** Ping-Yuan Wang, De-Zhu Li

**Affiliations:** 1 CAS Key Laboratory of Tropical Forest Ecology, Xishuangbanna Tropical Botanical Garden, Chinese Academy of Sciences, Menglun, Mengla, Yunnan 666303, China Xishuangbanna Tropical Botanical Garden, Chinese Academy of Sciences Menglun China; 2 University of Chinese Academy of Sciences, Beijing 100049, China University of Chinese Academy of Sciences Beijing China; 3 Germplasm Bank of Wild Species, Kunming Institute of Botany, Chinese Academy of Sciences, Kunming, Yunnan 650201, China Kunming Institute of Botany, Chinese Academy of Sciences Kunming China

**Keywords:** *
Dendrocalamus
*, woody bamboo, Poaceae, Yunnan, taxonomy

## Abstract

*Dendrocalamus menghanensis* P.Y.Wang & D.Z.Li, a new species of woody bamboos endemic to south Yunnan, China, is described and illustrated. The new species is morphologically similar to *D. semiscandens* and *D. birmanicus* but differs in having a reflexed culm sheath blade, 10 mm high culm sheath ligule, 1 mm high leaf sheath ligule, 4 florets and 1 glume.

## Introduction

The genus *Dendrocalamus* was described by Nees von Esenbeck (1835) and currently comprises of more than 50 species in tropical and subtropical regions of Asia (Ohrnberger 1999, Bamboo Phylogeny Group 2012). Several new species in this genus have been continuously described in recent years (Yang et al. 2016, Wang et al. 2016, Nguyen et al. 2017a, 2017b). There are about 30 species of *Dendrocalamus* distributed in China (including new species described in recent years) (Li et al. 2006). It is a typical paleotropical woody bamboo genus belonging to the subtribe BambusinaePresl (1830) of tribe Bambuseae Kunth ex Dumortier (1829). Within this subtribe, the three major genera are *Bambusa* (von Schreber 1789), *Dendrocalamus* and *Gigantochloa* Kurz ex Munro (1868). They formed a clade known as the BDG complex (Goh et al. 2010, 2013), also named “core Bambusinae”, but the long-standing problems for taxonomic delimitation and evolutionary relationships within the BDG complex have not been satisfactorily resolved (Goh 2012, Chokthaweepanich 2014, Zhou et al. 2017).

Most of the species of *Dendrocalamus* can be recognised by their thick-walled culms, swollen nodes reflexed culm sheath blade and aerial roots at the lower nodes. The species usually have white, blackish or light-brown hairs on the culm sheaths (Dransfield 1980). Compared to *Dendrocalamus*, it is easy to classify the *Bambusa* species by the erect culm sheath blade and conspicuous auricles and *Gigantochloa* by connate filaments. While checking the bamboos cultivated in Xishuangbanna Tropical Botanical Garden (XTBG), Chinese Academy of Sciences (CAS), we discovered an extraordinary *Dendrocalamus* species. The floret of this species has no lodicule, one plumose stigma, six stamens and completely separate filaments, indicating that it belongs to *Dendrocalamus* rather than to *Gigantochloa* or *Bambusa* (Li and Hsueh 1988a, 1988b, Dransfield and Widjaja 1995, McClure 1966, Wong 1995, Li et al. 2006, Clayton et al. 2008, Sungkaew 2008).

This new species resembles *D. semiscandens* (Li and Hsueh 1989) and *D. birmanicus*Camus (1932) in some morphological characters as discussed below (see Table 1). It was introduced in XTBG from Menghan Township, Jinghong, Yunnan, China in 1980.

## Material and methods

All measurements of the new *Dendrocalamus* species were taken from dried herbarium specimens and living individuals at XTBG, Menglun, Mengla, Yunnan province. For morphological characterisation, vegetative parts of plant material were measured using the living plants and the reproductive parts were analysed under an automated digital microscope (ZEISS Smartzoom 5) linked with a computer in Xishuangbanna Station for Tropical Rainforest Ecosystem Studies of XTBG, CAS. The morphological terminology followed McClure (1966).

## Taxonomy

### Dendrocalamus menghanensis

Taxon classificationPlantaePoalesPoaceae

P.Y.Wang & D.Z.Li, sp. nov. “勐罕龙竹” (Meng Han Long Zhu)

F3BE1D58F04C581785431BF243AD9DA1

urn:lsid:ipni.org:names:60479347-2

Figures 1 , 2

#### Type.

CHINA. Yunnan: Xishuangbanna, Menglun, 21°55.949'N, 101°15.139'E, 570 m alt., 18 November, 2010, *P.Y. Wang C130022* (holotype: HITBC!; isotype: KUN!).

#### Diagnosis.

*Dendrocalamus menghanensis* is morphologically similar to *D. semiscandens* and *D. birmanicus*, but can be easily distinguished from them by having a reflexed culm sheath blade, 10 mm high culm sheath ligule, 1 mm high leaf sheath ligule, 4 florets and 1 glume.

**Figure 1.**
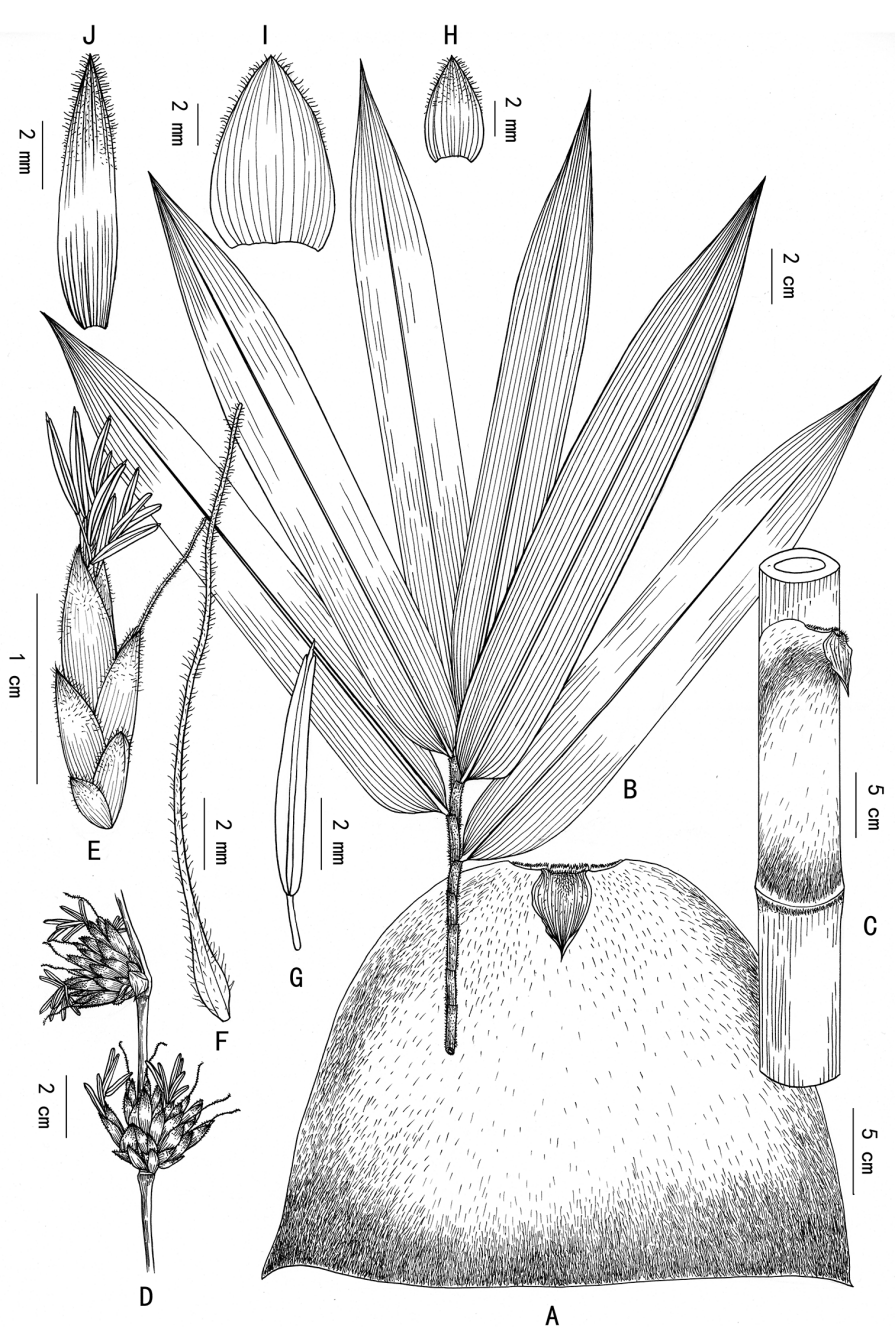
*Dendrocalamus menghanensis* P.Y. Wang & D.Z. Li. **A** Culm sheath (abaxial view) **B** ultimate branchlet with leaves **C** portion of young culm with culm sheath **D** portion of flowering branch **E** pseudo-spikelet **F** pistil **G** stamen **H** glume **I** lemma **J** Palea. Drawn from the holotype.

**Figure 2.**
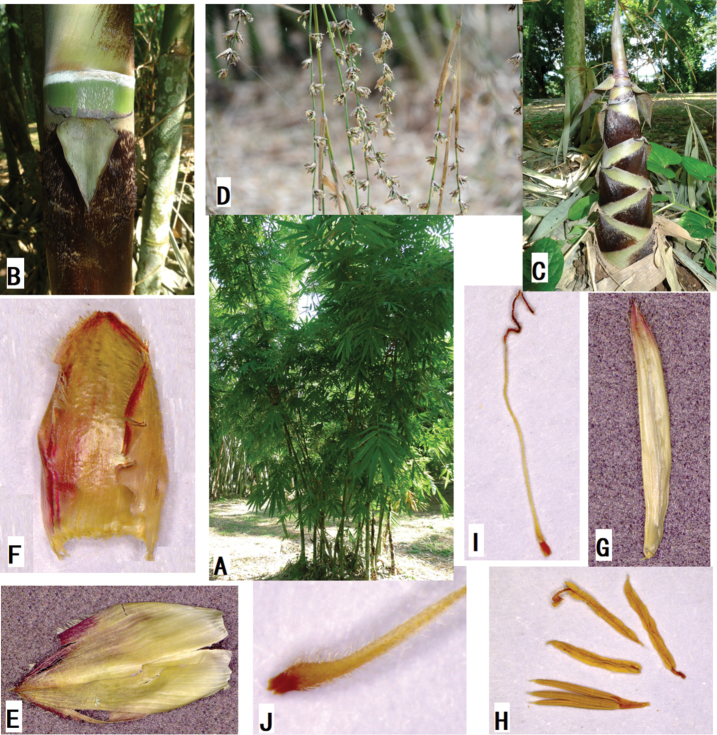
*Dendrocalamus menghanensis* P.Y.Wang & D.Z.Li. **A** Clump **B** portion of young culm with culm sheath **C** new shoot **D** flowering branches **E** glume **F** lemma **G** palea **H** floret **I** pistil **J** ovary.

#### Description.

Arborescent bamboo, perennials; rhizomes pachymorph, short necked. Culms apically pendulous, 8–12 m tall, 4–8 cm in diam.; culms terete, with a ring of white tomenta below sheath scars, internodes 20–40 cm long, wall 1–3.5 cm thick and almost solid at the base of culms; culm surface initially densely covered with white hairs and becoming glabrous later; culm sheaths deciduous, thickly leathery, 1/2 as long as the internodes, covered with dense brownish-black hairs, pale green initially, later becoming yellowish-brown with age; blades lanceolate, reflexed; auricles small inconspicuously lobed, oral setae absent; ligules ca. 10 mm high, dentate. Branching from lower nodes ca. 0.5–1.0 m above ground, branches several, usually subequal, sometimes 1 dominant; ultimate branchlets with 10–16 leaves, usually 12 leaves. Foliage leaves lanceolate, 11–30(-35) cm × 2–4.5(-6) cm, adaxial surface green and glabrous, abaxial surface pale green and pubescent, margins serrulate, secondary veins 7–11 pairs, usually 10 pairs, petioles 2–5 mm; leaf sheaths initially white hairy and later glabrous; auricles inconspicuous, ligules ca. 1 mm high, entire. Flowering branches pendulous, leafless, with clusters of 3 to 15(-60) pseudo-spikelets at each node; clusters 1–3.5 cm in diam.; pseudo-spikelets ovate-lanceolate, pale green, apically acute and light purple, 12–16 × 3–4 mm; fertile florets usually 4 per pseudo-spikelet; glumes 1, broadly ovate, 5–7 × 4–6 mm, margins ciliate at upper half; lemma ovate, 8–12 × 4–7 mm, pubescent, many-veined, apex mucronate, margins ciliate; palea oblanceolate, 2-keeled, 7–11 × 1–2 mm, keels and margins long ciliate; lodicules absent; stamens 6, ca. 6 mm long, ovary ovoid, pistil ca. 16 mm long, anthers yellow, filaments free, ca. 14 mm long; stigma 1, purple, plumose. Fruit unknown.

#### Distribution.

*Dendrocalamus menghangensis* is only known from Menghan Township, Jinghong, Yunnan, China.

#### Conservation status.

As a great many forests have been destroyed by local people in the last 30 years, we did not find the new species at the locality where it was introduced. Further investigation is required to find more distribution localities and determine the conservation status of the new species. At present, we consider it as DD (Deficient Data) according to the IUCN parameters (IUCN 2012).

#### Etymology.

The specific epithet refers to the original place of the new species, i.e. Menghan Town, Xishuangbanna, south Yunnan, China.

#### Phenology.

Shooting from July to October and flowering from December to May of the next year.

#### Additional specimens examined (paratype).

CHINA. Yunnan, Xishuangbanna, Menglun, 21°55.949'N, 101°15.139'E, 570 m alt., 7 December, 2010, *P.Y. Wang* C130051 (paratype: HITBC!, KUN!)

## Discussion

*Dendrocalamus menghanensis* is morphologically similar to *D. semiscandens* and *D. birmanicus*. However, the new species differs from them by having a reflexed culm sheath blade, 10 mm high culm sheath ligule, 1 mm high leaf sheath ligule, 4 florets and 1 glume. The major differences amongst these species are listed in Table 1. This new species is only found in Xishuangbanna which is located in one of the world’s biodiversity hotspots (Indo-Burma) (Myers et al. 2000). Many forests have been destroyed because of the plantation of rubber trees in this region in the past 30 years. Many species may become extinct before we know that they exist in Xishuangbanna. More field investigations need to be conducted in this region in future.

**Table 1.** Morphological differences amongst *Dendrocalamus menghanensis*, *D. semiscandens* and *D. birmanicus*.

Characters	*D. menghanensis*	*D. semiscandens*	*D. birmanicus*
Diameter of culm	4–8 cm	6–15 cm	ca. 8 cm
Culm sheath blade	reflexed	erect	reflexed
Number of florets	4	4–5	2–3
Culm sheath	covered with dense brownish-black hairs	covered with dark brown hairs	covered with dark brown hairs
Culm sheath ligule	10 mm	1 mm	3–4 mm
Leaf sheath ligule	1 mm	3–5 mm	1 mm
Glume	1	1–3	2
Anther	6 mm, yellow	3.7 mm, yellow, anther tip purple	3–4 mm

## Supplementary Material

XML Treatment for Dendrocalamus menghanensis
